# Parental administration of antipyretics to children with upper respiratory tract infections without consultation with a physician

**DOI:** 10.3325/cmj.2011.52.48

**Published:** 2011-02

**Authors:** Tea Andabaka, Tina Globočnik, Dalibor Vukelić, Susanna Esposito, Bruno Baršić

**Affiliations:** 1 Ewopharma Ltd., Zagreb, Croatia; 2 Dr Fran Mihaljević Hospital for Infectious Diseases, Zagreb, Croatia; 3 University of Zagreb School of Medicine, Zagreb, Croatia; 4 Department of Maternal and Pediatric Sciences, Università degli Studi di Milano, Fondazione IRCCS Ca' Granda Ospedale Maggiore Policlinico, Milan, Italy

## Abstract

**Aim:**

To evaluate the administration of antipyretics to children with upper respiratory tract infections (URTI) by their parents or guardians without consultation with physicians, and compare epidemiological and clinical characteristics of patients who received antipyretics and of untreated patients.

**Methods:**

A prospective observational study was performed in three pediatric clinics in Zagreb, Croatia, from March to June 2002. A total of 171 children aged from 2 to 14 years with symptoms and signs of URTI lasting more than 2 days and fever above 38°C lasting more than 2 days were included in the study. Data were collected on the usage of antipyretics, patients’ demographic and epidemiological characteristics, and clinical signs and symptoms.

**Results:**

Antipyretics, predominantly paracetamol, were used in 29.8% of patients. Their usage was less frequent in children attending day-care centers (49% of treated and 70% of untreated children, *P* = 0.014) and in children with reiterated URTIs (33.3% of treated and 55.8% of untreated children, *P* = 0.008). However, it was more frequent in children with recent URTIs in the family (33.3% of treated and 7.5% of untreated children, *P* < 0.001). Overall, most clinical signs and symptoms of URTI were notably less pronounced in patients treated with antipyretics.

**Conclusions:**

Antipyretics use correlated with less pronounced clinical signs and symptoms of infection, which indicates their anti-inflammatory activity, but also with negative effects such as lethargy. It is necessary to educate parents on the positive and negative aspects of antipyretics use and on the optimal choice of an antipyretic drug.

Infections of the upper respiratory tract (URTI) are associated with some of the most common infectious diseases, like pharyngitis, rhinitis, sinusitis, and otitis media, which account for millions of visits to physicians annually. URTIs, mostly caused by respiratory viruses, are the most frequent illnesses in childhood, often characterized by a rapid onset of fever (1).

Fever can enhance host resistance to infection, and interventions to reduce fever may negatively affect the outcome of the illness (2-4). Additionally, fever is, particularly in children, associated with irritability, stress, and high parental anxiety. Parental fear about the presumed harmful effects of fever in children (also called “fever phobia”) is still common, and in most cases is caused by misconceptions (5). Most caregivers and many clinicians erroneously believe that treatment of fever will relieve symptoms and prevent harmful effects such as febrile convulsions (6).

The drugs most commonly used for treating fever are paracetamol (acetaminophen), aspirin (acetylsalicylic acid), and ibuprofen (7). As antipyretic drugs, paracetamol and ibuprofen are also approved for treating fever in children. Current World Health Organization guidelines on the management of fever recommend the use of paracetamol for treating children with fever ≥39°C (8). However, recent studies show that the excessive use of paracetamol might be associated with greater morbidity (asthma, allergic rhinitis, and eczema) (9-12).

URTIs are the most common reason for the administration of antipyretics to children, which is often done without supervision of a physician (13).

Our research was performed as a part of a larger clinical study that investigated the role of a multiplex polymerase chain reaction method for the detection of respiratory bacteria in children with URTI (14). The aim of our research was to assess the administration of antipyretics to children with URTIs by their parents or guardians and without consultation with physicians, and to compare the demographic, epidemiological, and clinical characteristics in patients who received antipyretics and those who did not.

## Patients and methods

This research was done as a part of a larger prospective observational clinical study on the role and prevalence of *Streptococcus pneumoniae*, *Moraxella catarrhalis*, *Hemophylus influenzae*, *Chlamydia pneumonia,* and *Mycoplasma pneumoniae* in children with URTI. The study was performed by 8 attending physicians at 3 sites in Zagreb (University Pediatric Hospital Zagreb, Dr Fran Mihaljević University Hospital for Infectious Diseases, and Primary Care Health Center Trnje) in accordance with the ethical standards set by the institutional Ethics Committees. We planned to include a sample of 200 children, in order to estimate the incidence of atypical pathogens. Each physician was provided with customized forms for entering data on the children eligible for inclusion into the study.

Children eligible for inclusion were those aged 2 to 14 years, with symptoms and signs consistent with URTI lasting for more than 2 days, fever above 38°C lasting for more than 2 days, no antibiotic treatment received, and informed consent signed by their parents or guardians. The same child could not have participated in the study twice during the period of enrollment, from March to June 2002. All the patients included in hospitals were treated as outpatients and did not differ in the intensity of symptoms from the patients included in the study at the primary care health center.

For each participant, we collected demographic and epidemiological data (sex, age, body weight and height and data on attendance of school or day-care centers, siblings, family history of URTI, prior episodes of URTI, chronic diseases, allergies, concomitant medications, and duration of symptoms), bacteriological findings, and clinical characteristics of URTI (sudden or gradual onset of disease, presence of non-specific findings like lethargy, irritability, anorexia, vomiting, chills and headache, cough, nose obstruction, quality of nasal discharge, ear pain, ear drainage, presence of ear fluid, middle ear effusion, hyperemic or transparent tympanic membrane, enlarged cervical lymph nodes, throat erythema, throat exudates, and throat edema). The attending physician recorded the presence or absence of specific and non-specific clinical findings in every child.

During the first visit, parents were asked whether they had administered any medication to their child for the current episode of URTI before visiting the physician. They were asked to provide data on the type of drug, route of administration, dose, and reason for usage. Patients were divided into two groups: the group that had received any dose of antipyretics and the group that had not received antipyretics for the current URTI episode before they visited a physician.

### Statistical analysis

The collected data were entered into a database that had been developed using the validated software Softleks DDE (Softleks d.o.o., Zagreb, Croatia). All data were entered twice and all discrepancies were resolved by examining the original data collection forms. For the purpose of logistic regression analysis and in order to maintain the power of this research, some missing values were imputed. This imputation was based on the variables clinically related to the variable with the missing values. Where such practice was not possible, a missing value was replaced with “no,” based on the presumption that physicians would omit the parameter’s value if the characteristics were not present. The missing continuous variables were imputed by median values. Bivariate analysis did not include the imputed values (15).

Results of univariate analysis were expressed as median and interquartile range (25th-75th percentile) for continuous variables and as frequencies and percents for categorical variables. Bivariate analysis, used for comparing the two groups, was performed using the Wilcoxon test for continuous variables, χ^2^ test for categorical variables, and Fisher exact test when appropriate. Logistic regression analysis was performed to identify independent factors associated with the use of antipyretics as self-administered medication. Variables that differed between the two groups in bivariate analysis (*P* < 0.050) were included into the model. The assessment of the fit of the statistical model was done by the Hosmer-Lemeshow goodness-of-fit test. All *P* values are two-tailed, with the significance level set at 0.05. All the analyses were performed using the SAS 9.1 statistical software (SAS Institute Inc., Cary, NC, USA).

## Results

The study population consisted of 172 patients. For one child, no written information was available, so the data were analyzed for 171 children who were included in the study based on the inclusion and exclusion criteria. The planned study population of 200 children was not reached due to the low frequency of infections in the study period.

Before the inclusion into the study, 56 children (32.8%) had received medication for the symptomatic treatment of a URTI episode without physician’s supervision. Most commonly administered drugs were antipyretics, used in 51 patients (29.8%). Forty-two patients were given paracetamol orally (24.6%), 5 were given acetylsalicylic acid (2.9%), and 7 nonsteroidal anti-inflammatory drugs (4.1%) (Table 1). For the purpose of all further analyses, patients were divided into two groups: one group that had received antipyretics (51 patients) and the other that had not (120 patients). Since the data on the administered antipyretic drugs dosage were not reliably collected, they were not used in the analyses. Other drugs taken as symptomatic treatment were butamirate applied orally in 4 children (2.3%) to alleviate cough and loratadin in 2 patients (1.2%) as an antihistaminic drug. Tobramycin was administered topically to one patient. As the systemic effect of antibiotics is not to be expected after the topical administration, the data on this patient were retained in the analysis.

**Table 1:** Parental administration of medication to children with symptoms of upper respiratory tract infections (URTI) before visiting a physician

Medication	No. (%) of patients
Any therapy for symptomatic treatment of URTI	56 (32.8)
Antipyretic drugs:	51 (29.8)
only paracetamol	39 (22.8)
only acetylsalicylic acid	5 (2.9)
only non-steroid anti-inflammatory drugs	4 (2.3)
combined (paracetamol + ibuprofen and/or diclofenac)	3 (1.8)

The majority of patients were preschool children (Table 2). Eight of 171 patients reported that they suffered from chronic diseases (eg, asthma, epilepsy, bronchitis, etc.), and 2 of them were regularly taking concomitant medications. A higher incidence of allergies was reported in the group of patients who had received antipyretics (Table 2). Out of 12 patients in whom allergy was reported, 8 reported using agents causing allergy. Three children were allergic to penicillin, 2 to dust, 2 to pollens, and 1 to feathers and birch.

**Table 2:** Comparison of demographic and epidemiologic data on patients who did and did not receive antipyretic drugs for the upper respiratory tract infection (URTI) before visiting a physician

	No. (%) of patients		
Characteristic	treated with antipyretics (n = 51)	not treated with antipyretics (n = 120)	*P*
Age, years, median (range)	6.0 (3.0-9.0)	5.0 (3.5-7.0)	0.360*
Age group			
2-3 y	13 (25.5)	27 (22.5)	0.457^†^
3.1-6.9 y	19 (37.3)	57 (47.5)	
≥7 y	19 (37.3)	36 (30.0)	
Sex:			
male	27 (52.9)	70 (58.3)	0.613^‡^
female	24 (47.1)	50 (41.7)	
Chronic diseases	2 (3.9)	6 (5.0)	1.000^‡^
Allergies	7 (13.7)	5 (4.2)	0.044^‡^
Attending day-care center	25 (49.0)	84 (70.0)	0.014^‡^
Attending school	19 (37.3)	35 (29.2)^§^	0.369^‡^
Having siblings	30 (58.8)^§^	79 (65.8)	0.391^‡^
Having sister(s)	16 (31.4)	60 (50.0)	0.029^‡^
Having brother(s)	18 (35.3)	41 (34.2)	0.999^‡^
Recent URTI in family	17 (33.3)^§^	9 (7.5)	<0.001^‡^
Frequent URTI	17 (33.3)	67 (55.8)	0.008^‡^
Sudden onset of URTI episode	27 (52.9)	60 (50.0)	0.741^‡^
Duration of symptoms, days, median (range)	3.0 (2.0-4.0)	3.0 (3.0-3.0)^║^	0.275*

*Wilcoxon test.

†χ^2^ test.

‡Fisher exact test.

§Data missing for 1 patient.

║Data missing for 4 patients.

Attendance of a day-care center increased the risk of frequent episodes of URTI (odds ratio [OR], 2.67; 95% confidence interval [CI], 1.40-5.12). Children who attended day-care centers (*P* = 0.005) and those who had frequent URTIs (*P* = 0.008), defined as at least 3 episodes of URTI in 6 months preceding the enrollment, were given antipyretics less frequently (Table 2). On the other hand, children with a recent URTI episode in the family were given antipyretics more frequently (Table 2).

Multivariate logistic regression analysis was performed to determine the epidemiologic factors that have the greatest influence on the parental tendency to administer antipyretics to their children. Three explanatory variables entered the model: attendance of day-care centers, frequent episodes of URTI, and a recent URTI in the family. Multivariate analysis showed that the history of frequent URTI in the child was associated with not using antipyretics (OR, 2.13; 95% CI, 1.02-4.48), while a recent URTI in the family significantly increased the probability of using antipyretics (OR, 5.82; 95% CI, 2.32-14.61).

In order to observe the differences in the clinical presentation of URTI, we compared the symptoms and signs of URTIs in two groups of patients. Patients who had not received antipyretics more frequently experienced irritability, vomiting, chills, headache, nasal speech, and cough (Table 3). Patients who had received antipyretics more frequently experienced lethargy, defined as daytime sleepiness (*P* = 0.029). Patients who had not received antipyretics more frequently experienced most clinical signs of respiratory infection (nasal obstruction, nasal discharge, non-transparent tympanic membrane, severe throat erythema, throat exudates, uvular edema and purulent exudates on tonsils) (Table 3). On the other hand, patients who had received antipyretics more frequently experienced ear fluid and pharyngeal edema (*P* = 0.020 and *P* = 0.005, respectively).

**Table 3:** Comparison of clinical signs and symptoms of upper respiratory tract infection (URTI) in patients who did and did not receive antipyretic drugs before visiting a physician

	No. (%) of patients		
Symptom or sign	treated with antipyretics (n = 51)	not treated with antipyretics (n = 120)	*P*
Lethargy	12 (23.5)	12 (10.0)^†^	0.029*
Irritability	27 (52.9)	85 (70.8)	0.034*
Anorexia	40 (78.4)	79 (65.8)	0.145*
Vomiting	11 (21.6)	46 (38.6)	0.035*
Chills	9 (17.6)	56 (46.7)	<0.001*
Headache	30 (58.8)	91 (75.8)	0.029*
Nasal speech	27 (52.9)^†^	100 (95.8)	0.006*
Nasopharyngeal irritation	42 (82.4)^†^	115 (90.8)	1.000*
Cough	32 (62.8)	95 (79.2)	0.035*
Ear pain	8 (15.7)	35 (29.2)	0.083*
Hearing loss	0	0^‡^	
Enlarged cervical lymph nodes	32 (62.8)	77 (64.2)	0.864*
Nasal obstruction	31 (60.8)	98 (81.7)^§^	0.006*
Nasal discharge:	29 (56.9)	111 (92.5)	<0.001*
serous	11 (21.6)	51 (42.5)	0.010*
seromucous	17 (33.3)	47 (39.2)	0.495*
mucopurulent	2 (3.9)	18 (15.0)	0.040*
Ear drainage	0	1 (0.8)^†^	1.000*
Presence of fluid	3 (5.9)	0^§^	0.020*
Hyperemic tympanic membrane	11 (21.6)	23 (19.2)^†^	0.834*
Transparent tympanic membrane	38 (74.5)	39 (32.5)^†^	<0.001*
Middle ear effusion	3 (5.9)	1 (0.8)^║^	0.080*
Erythema of the throat:	51 (100)	112 (93.3)	0.999*
absent	0 (3.6)	8 (6.7)	
mild	10 (19.6)	35 (29.2)	
moderate	37 (72.6)	60 (50.0)	
severe	4 (7.8)	17 (14.2)	
Exudate in the throat:	19 (37.3)	81 (67.5)	<0.001*
seromucous	13 (68.4)	65 (80.2)	
mucopurulent	6 (31.6)	16 (19.8)	0.263*
Uvular edema	7 (13.7)	82 (68.3)	<0.001*
Pharyngeal edema	44 (86.3)	78 (65.0)^§^	0.005*
Palatal edema	28 (54.9)	61 (50.8)^†^	0.738*
Tonsils out of palatal arches	37 (72.6)^†^	67 (55.8)^†^	0.059*
Swollen tonsils	38 (74.5)^†^	70 (58.3)	0.057*
Purulent exudates on tonsils	5 (9.8)^†^	42 (35.0)^†^	<0.001*

*Fisher exact test.

†Data missing for 1 patient.

‡Data missing for 9 patients.

§Data missing for 2 patients.

║Data missing for 6 patients.

The connection between the use of antipyretics and the incidence of symptoms and signs of infection was evaluated by a multivariate analysis. In order to assess the factors independently associated with the use of antipyretics, two models were formed. The first model included 6 explanatory variables representing symptoms of URTIs: irritability, lethargy, vomiting, chills, headache, and cough. Multicollinearity was not observed. There were no suspected interactions. The model fitted well (Hosmer-Lemeshow goodness-of-fit test: χ^2^ = 11.43, *P* = 0.671). Likelihood-ratio test did not confirm the global null hypothesis (*P* < 0.001). The use of antipyretics was independently associated with more than three times higher incidence of lethargy (OR, 3.37; 95% CI, 1.25-9.13) and inversely associated with the presence of chills (OR, 0.36; 95% CI, 0.15-0.91). The second model included 4 explanatory variables representing clinical signs on physical examination: presence of nasal discharge, presence of completely transparent tympanic membrane, presence of throat exudates, and pharyngeal edema. Uvular edema and purulent exudates on tonsils were omitted from the model because of the small number of patients with positive findings. Multicollinearity was not observed. There were no suspected interactions. The model fitted well (Hosmer-Lemeshow goodness-of-fit test: χ^2^ = 5.12, *P* = 0.644). Likelihood-ratio test did not confirm the global null hypothesis (*P* < 0.001). The presence of normal, transparent tympanic membrane on otoscopy was distinctly more common in children who had received antipyretics (OR, 5.50; 95% CI, 2.31-13.10), as was the absence of nasal discharge and throat exudates (inverse association between antipyretics use and nasal discharge/throat exudates, OR, 0.10; 95% CI, 0.04-0.29 and OR, 0.40; 95% CI, 0.17-0.90, respectively). On the other hand, the occurrence of pharyngeal edema was more than three times more common in patients who had received antipyretics (OR, 3.43; 95% CI, 1.24-9.45).

## Discussion

Our observational study showed that parents administered antipyretics to their children with acute URTI without consultation with physicians in less than one third of cases. These results are compatible with previous reports (5). The reasons why parents restrain the children’s intake of antipyretics were not further evaluated. The most commonly administered antipyretic was paracetamol, applied orally. Combined paracetamol plus ibuprofen therapy was administered in fewer than 2% of patients, which is in accordance with the recent guidelines (16). Almost 3% of parents administered aspirin, which indicates a lack of awareness of the potential hazard associated with the use of acetylsalicylic acid (17).

Even though patients who had received antipyretics had a higher incidence of allergies, this can hardly be associated with antipyretics use, because it was only recorded during an actual episode of URTI. It can only be assumed that the parent, who decided to administer antipyretics to the child during our study, had also been giving antipyretics regularly to his child during URTI episodes.

Our study showed that the parents’ tendency to administer antipyretics to their children was related to the history of frequent URTIs in their children and to day-care center attendance. Almost half of the patients registered for the study had a history of frequent URTI episodes, while almost two thirds were regularly attending day-care centers. Children attending day-care centers were shown to be at a higher risk of acquiring frequent URTIs (18) and were less likely to receive antipyretics. This finding suggests that previous experience of self-limiting illness leads to a lower consumption of antipyretics. It is also possible that parents of these children with time become reluctant to use antipyretics. In contrast, children with a recent case of URTI in their family received antipyretics more often. This might be related to their easy availability and storing of unused drugs in the household. However, these results are inconclusive because of the small number of patients with a history of a recent URTI in the family. Low exposure to URTI in the family also implies that the acquisition of URTI at home is rare.

Our study showed that the use of antipyretics correlated with the less pronounced group of clinical signs and symptoms of respiratory infection, which implies the drugs’ anti-inflammatory activity. However, antipyretics use was also associated with a greater incidence of lethargy, suggesting that antipyretics could cause certain unfavorable outcomes. However, lethargy might be the consequence of prior irritability and lack of sleep in the initial phase of the disease. Other drugs which might cause lethargy were not concomitantly used. Besides this, our results should be taken with caution since a study of this design cannot prove the causal relationship between the use of antipyretics and lethargy.

The primary indicators of the reduced intensity of inflammation in children receiving antipyretics were transparent tympanic membrane and absence of nasal discharge and throat exudates. Visible changes on the tympanic membrane were more than five times less common in patients receiving antipyretics, probably due to the reduction in the signs of inflammation. Pharyngeal edema, on the other hand, occurred more often in patients taking antipyretics. However, these results are inconclusive due to the low small number of patients with such symptoms.

Overall, the lower presence and intensity of clinical signs could hardly be attributed to the activity of nonsteroidal anti-inflammatory drugs or acetylsalicylic acid, because these drugs had been given to less than a quarter of patients treated with antipyretics. Reduction in signs of inflammation was most probably caused by paracetamol use, even though a Cochrane review reported inconsistent and weak evidence to support this view (19).

Our study has several limitations. This was an exploratory analysis that was not predefined by the protocol. Additional parameters, which would help interpret the observed differences in the two groups of patients, were not investigated, and a selection bias is present. Another drawback is that we did not properly collect the data on the dosages of antipyretics administered to children. It was unclear whether the values in the collection forms were daily or total amounts, and therefore these data had to be omitted from the analyses. For the purpose of logistic regression analysis, some missing values were imputed. Since the number of missing values is relatively small, imputation did not significantly change the results. Notwithstanding these limitations, our study undoubtedly indicates some beneficial effects of paracetamol use, which cannot easily be diminished by some potentially negative long term effects like the incidence of asthma and allergies (9-12). However, the controversies regarding the use of paracetamol in children remain, and further studies are needed to investigate these (19).

Our study revealed different parents’ approaches to the use of antipyretics, probably based on previous experience and misconceptions, and not on medical facts. Therefore, it is necessary to educate parents about both positive and negative aspects of antipyretics use and the optimal choice of an antipyretic drug. Parents should always weigh both beneficial and possible long term detrimental effects before administering antipyretics to their children. Recent guidelines recommend the use of antipyretics in children only when fever is associated with evident discomfort (20). High use of antipyretics makes further studies on this topic justified and indispensable.

## References

[1] Fairchok MP, Martin ET, Chambers S, Kuypers J, Behrens M, Braun LE (2010). Epidemiology of viral respiratory tract infections in a prospective cohort of infants and toddlers attending daycare.. J Clin Virol.

[2] Kramer MS, Naimark LE, Roberts-Bräuer R, McDougall A, Leduc DG (1991). Risks and benefits of paracetamol antipyresis in young children with fever of presumed viral origin.. Lancet.

[3] Kluger MJ (1992). Drugs for childhood fever.. Lancet.

[4] Roberts NJ (1991). Impact of temperature elevation on immunologic defenses.. Rev Infect Dis.

[5] Crocetti M, Moghbeli N, Serwint J (2001). Fever phobia revisited: have parental misconceptions about fever changed in 20 years?. Pediatrics.

[6] van Stuijvenberg M, Derksen-Lubsen G, Steyerberg EW, Habbema JDF, Moll HA (1998). Randomized controlled trial of ibuprofen syrup administered during febrile illness to prevent febrile seizure recurrences.. Pediatrics.

[7] Autret E, Reboul-Marty J, Henry-Launois B, Laborde C, Courcier S, Goehrs JM (1997). Evaluation of ibuprofen versus aspirin and paracetamol on efficacy and comfort in children with fever.. Eur J Clin Pharmacol.

[8] World Health Organization. Integrated management of childhood illness. Geneva (Switzerland): World Health Organization; WHO/FCH/CAH/ 2000.00.12. Available from: http://www.emro.who.int/cah/pdf/IMCI-Adaptation-Yem.pdf*.* Accessed: February 7, 2011.

[9] Karimi M, Mirzaei M, Ahmadieh MH (2006). Acetaminophen use and the symptoms of asthma, allergic rhinitis and eczema in children.. Iran J Allergy Asthma Immunol.

[10] Beasley R, Clayton T, Crane J, von Mutius E, Lai CK, Montefort S (2008). Association between paracetamol use in infancy and childhood, and risk of asthma, rhinoconjunctivitis, and eczema in children aged 6-7 years: analysis from Phase Three of the ISAAC programme.. Lancet.

[11] Barr RG (2008). Does paracetamol cause asthma in children? Time to remove the guesswork.. Lancet.

[12] Cohet C, Cheng S, MacDonald C, Baker M, Foliaki S, Huntington N (2004). Infections, medication use, and the prevalence of symptoms of asthma, rhinitis, and eczema in childhood.. J Epidemiol Community Health.

[13] Das B, Sarkar C, Majumder AG (2006). Medication use for pediatric upper respiratory tract infections.. Fundam Clin Pharmacol.

[14] Geertsen R, Kaeppeli F, Sterk-Kuzmanovic N, Andrasevic S, Anic-Milic T, Dobec M (2007). A multiplex PCR assay for the detection of respiratory bacteriae in nasopharyngeal smears from children with acute respiratory disease.. Scand J Infect Dis.

[15] Refaat M. Treatment of missing values. In: Data preparation for data mining using SAS. Amsterdam: Morgan Kaufman; 2007. p.171-206.

[16] Richardson M, Lakhanpaul M, Guideline Development Group and the Technical Team (2007). Assessment and initial management of feverish illness in children younger than 5 years: summary of NICE guidance.. BMJ.

[17] Beutler AI, Chesnut GT, Mattingly JC, Jamieson B (2009). FPIN's Clinical Inquiries. Aspirin use in children for fever or viral syndromes.. Am Fam Physician.

[18] Bellanti JA (1997). Recurrent respiratory tract infections in paediatric patients.. Drugs.

[19] Meremikwu M, Oyo-Ita A (2002). Paracetamol for treating fever in children.. Cochrane Database Syst Rev.

[20] Chiappini E, Principi N, Longhi R, Tovo PA, Becherucci P, Bonsignori F (2009). Management of fever in children: summary of the Italian Pediatric Society guidelines.. Clin Ther.

